# Effects of rat anti-VEGF antibody in a rat model of corneal graft rejection by topical and subconjunctival routes

**Published:** 2011-01-11

**Authors:** Nicolas Rocher, Francine Behar-Cohen, Jean-Antoine C. Pournaras, Marie-Christine Naud, Jean-Claude Jeanny, Laurent Jonet, Jean-Louis Bourges

**Affiliations:** 1 Université Paris Descartes; Faculté de Médecine, Assistance Publique des Hôpitaux de Paris, Hôtel-Dieu Hospital, Department of Ophthalmology, Paris, France; 2 INSERM UMRS 872-17, Centre de Recherche des Cordeliers, Paris, France; 3 Laboratory of Ocular Vascular Diseases, Faculty of Medicine, University of Geneva, Geneva, Switzerland

## Abstract

**Purpose:**

To compare the effect of a rat anti-VEGF antibody, administered either by topical or subconjunctival (SC) routes, on a rat model of corneal transplant rejection.

**Methods:**

Twenty-four rats underwent corneal transplantation and were randomized into four treatment groups (n=6 in each group). G1 and G2 received six SC injections (0.02 ml 10 µg/ml) of denatured (G1) or active (G2) anti-VEGF from Day 0 to Day 21 every third day. G3 and G4 were instilled three times a day with denatured (G3) or active (G4) anti-VEGF drops (10 µg/ml) from Day 0 to Day 21. Corneal mean clinical scores (MCSs) of edema (E), transparency (T), and neovessels (*nv*) were recorded at Days 3, 9, 15, and 21. Quantification of neovessels was performed after lectin staining of vessels on flat mounted corneas.

**Results:**

Twenty-one days after surgery, MCSs differed significantly between G1 and G2, but not between G3 and G4, and the rejection rate was significantly reduced in rats receiving active antibodies regardless of the route of administration (G2=50%, G4=66.65% versus G1 and G3=100%; p<0.05). The mean surfaces of neovessels were significantly reduced in groups treated with active anti-VEGF (G2, G4). However, anti-VEGF therapy did not completely suppress corneal neovessels.

**Conclusions:**

Specific rat anti-VEGF antibodies significantly reduced neovascularization and subsequent corneal graft rejection. The SC administration of the anti-VEGF antibody was more effective than topical instillation.

## Introduction

Vascular endothelial growth factor (VEGF) promotes angiogenesis in blinding eye diseases, such as retinopathy of prematurity, age-related macular degeneration (AMD), and diabetic retinopathy [1]. Anti-VEGF strategies have changed the prognosis of neovascular AMD [2,3].

While anti-VEGF antibodies have not received FDA approval to treat neovessels growing in the anterior segment of the eye, it has been suggested they can help treat pterygium [4], herpetic keratitis [5],and Stevens-Johnson syndrome [6] via topical or subconjunctival (SC) administration. In such indications, anti-VEGFs are used at less than 100 times the systemic doses used to treat colorectal cancer, thereby limiting the risk of systemic side effects [7,8].

Anti-VEGF treatment administrated by the topical [9] or SC route [10] has also been shown to reduce corneal neovascularization in wound healing models in which neovessels are generated by corneal injury [11] or by limbal stem cell deficiency [12].

In corneal transplantation, preexisting vascularization of the corneal bed is an important risk factor for immune rejection [13-15]. The reduction of preexisting neovessels using anti-VEGF has been shown to improve the success of high-risk allogeneic corneal transplantation in animal models [16-18]. Therefore, several attempts have been made to prevent or reduce corneal rejection using local anti-VEGF therapies in animal models and in humans [19]. However, no definite conclusions can be drawn from animal studies because bevacizumab, a humanized anti-VEGF antibody that binds poorly to murine VEGF-A, has typically been used. Indeed, to neutralize murine VEGF-A, a 1,000 times higher concentration of bevacizumab is required; it also dissociates faster [20,21]. Moreover, it remains unclear whether SC injections or topical administration is preferable in this indication [12,22]. To address these questions, we have evaluated the effects of a rat anti-VEGF antibody, administered either by SC injection or topical instillation, on corneal rejection in a rat model.

## Methods

All animal studies complied with the European Community Standard of Care and Use of Laboratory Animals and the Association for Research in Vision and Ophthalmology (ARVO) Statement for the Use of Animals in Ophthalmic and Visual Research. Protocols were approved by the ethical committee of Paris Descartes University.

### Animal model

Ten week-old Brown Norway (BN) females rats and Lewis male rats (n=24) were obtained from Elevage Janvier (Le Genest Saint Isle, France). The Lewis rats were anesthetized with a mixture of 125 mg/kg ketamine chlorhydrate (UVA, Ivry-sur-Seine, France) and 5 mg/kg chlorpromazine (Specia Rhône Poulenc, Paris, France). Each animal was systematically weighed before examination and/or experimentation procedures. For corneal transplantation, penetrating keratoplasty (PK) was performed by two corneal surgeons (J.L.B. and N.R.), as previously described [23]. In brief, corneal buttons from the sacrificed BN rats were obtained using a 3.0 mm trepan and were grafted into a 3.0 mm corneal bed in the right eyes of the Lewis rats. The day of surgery was Day 0. Paracentesis was performed before trephination under maximum pupil dilation (tropicamide, Théa, Clermont-Ferrand, France) and the anterior chamber was filled with viscoelastic fluid (Healonid, Pharmacia, Uppsala, Sweden). A 3.0 mm trephination was performed using a biopsy punch and was completed with Vanas scissors. The BN corneal button was secured in place by a 10–0 monofilament 8-path running suture (Ethicon, Saint Stevens-Woluwe, Belgium) with a buried suture knot to limit artifactual vascular growth. No treatment was applied at the end of surgery other than the tested one. In this model, the rejection process was initiated on Day 5 and was completed by Day 14 after PK [24]. Transplanted eyes with intraoperative or immediate post-surgical complications before Day 2 (suture rupture, endophthalmitis, cataract, iris herniation) were excluded and replaced by the next grafted animal on the randomization schedule.

### Neutralizing VEGF agent

We used a 150 kDa rat anti-VEGF antibody (R & D Systems, Minneapolis, MN) neutralizing to rrVEGF164, rmVEGF120, rmVEGF164, rhVEGF121, or rhVEGF165, with a less than 2% cross-reaction with VEGF B, C, and D. The antibody was aseptically diluted with PBS (Dulbecco Sigma-Aldrich, Lyon, France) at a dose of 10 µg/ml. The necessary volume of anti-VEGF treatment was prepared for SC injection and topical instillation in aliquots every day.

As a control, non-immune VEGF antibody was prepared in the same way and was further denatured by heating at 80 °C for 15 min.

### Subconjunctival injections

Prior to the SC injections, the rats were anesthetized with 1.5 mg/kg of chlorhydrate ketamine and 0.23 mg/kg of chlorpromazine. The SC injections were performed on the day of the surgical procedure and on Days 3, 6, 9, 12, 15, and 18 before sacrifice. To prevent trauma and take advantage of the slow release of the therapeutic agent, injections were not performed on a daily basis [12].

SC injections (2 µl) were performed in grafted eyes using a 29 1/2 gauge needle (micro-fine 0.3 ml SafetyGlide™ insulin syringe; Becton, Dickinson and Company, Franklin Lakes, NJ). Injections were performed under an optical microscope and were situated at least 2 mm away from the needle puncture. To reduce leakage, conjunctival punctures were pinched with a microsurgical forceps 3 s immediately after injection.

### Treatment protocol

After PK, the rats (n=24) were randomly assigned to four treatment groups, as follows. Group 1 (n=6) received an SC injection of 0.02 ml denatured 10 µg/ml anti-VEGF, repeated every third day, from Day 0 to Day 18. Group 2 (n=6) received an SC injection of 0.02 ml 10 µg/ml anti-VEGF, repeated every third day, from Day 0 to Day 18. Group 3 (n=6) was instilled with denatured 10 µg/ml anti-VEGF drops, three times a day, from Day 0 to Day 20. Group 4 (n=6) was instilled with 10 µg/ml anti-VEGF drops, three times a day, from Day 0 to Day 20. Grafted rats were sacrificed on Day 21 using an overdose of intraperitoneal pentobarbital (Ceva Santé Animal, Libourne, France).

### Evaluation of the rejection process

Grafted corneas were observed with a slit-lamp on Days 0, 3, 6, 9, 12, 15, 18, and 21 before treatment. Pictures of the graft were obtained at each time point. The progression of edema and transparency and the growth of neovessels both in the button area and in the recipient were scored by two masked examiners, as follows [25]. For corneal transparency: 0 (clear cornea), 1 (slight opacity), 2 (mild opacity with iris details visible), 3 (moderate, iris details not visible), and 4 (white cornea). For edema: 0 (no edema), 1 (slight edema), 2 (diffuse and moderate stromal edema), and 3 (diffuse marked stromal edema). For neovascularization: 0 (no observable growth of new vessels), 1 (new vessels invading less than 1/3 of the recipient bed), 2 (new vessels invading less than 2/3 of the recipient bed), 3 (new vessels growing up to the limiting ring of the graft), and 4 (new vessels invading the graft). A graft was rejected when opacity was greater than or equal to 3 [25], which is greater than the opacity seen in isografts [26].

### Corneal staining

On Day 21, the grafted rats were sacrificed, the eyes were enucleated, and the corneas were flat mounted in 4% paraformaldehyde for 1 h. Fixed flat mounted corneas were rinsed with PBS, then incubated for 20 min in 20 mM EDTA (Sigma-Aldrich Co., St. Louis, MO) to enhance the stromal penetration of lectin protein. Corneas were post-fixated for 1 min with iced acetone and rinsed two times with 1% triton X100/PBS. Corneas were exposed to 2% bovine albumin serum (BSA) for 1 h at room temperature and were then incubated with 1:100 TRITC conjugated *G. Simplicifolia* (Bandeiraea) isolectin (Sigma-Aldrich) in 1% triton X100/PBS overnight at 4 °C. Each slide was rinsed three times in PBS and mounted in PBS:glycerol (1:1).

After we ensured there was no autofluorescence, images of flat mounted corneas were captured using Olympus fluorescence microscopy (Olympus America Inc.,Center Valley, PA) at 550 nm and 20× magnification and were digitally stored for analysis. Image J software (Wayne Rasband, NIMH, Bethesda, MD) was used to conduct fluorescent morphometric analysis, which quantified the percentage of neovascularized corneas within the limbus boundaries as opposed to the total corneal area.

### Statistical analysis

Bodyweight modifications were assessed by comparing the mean bodyweights between time points using a Mann–Whitney test. Rejection scores, mean clinical scores (MCSs), and neovascularization areas were also compared using a Mann–Whitney non-parametric test, as each single treatment group was only compared to its related control throughout the analysis. For all comparisons, an alpha level of <0.05 was considered significant.

## Results

### Animals

Six animals were excluded within 2 days after the procedure (1 from Group 1, 2 from Group 2, 2 from Group 3, and 1 from Group 4) due to four cases of endophthalmitis (1 from Group 1, 2 from Group 2, and 1 from Group 3) and two discontinuations of the running suture (1 from Group 3 and 1 from Group 4). Each animal was replaced following the randomization schedule. Before experimentation, the average weight of grafted rats from Groups 1, 2, 3, and 4 was 321.5 g, 312.9 g, 315.6 g, and 322.9 g; these did not change much throughout the study (p>0.1).

### Clinical evaluation of the rejection process

**Figure 1:**
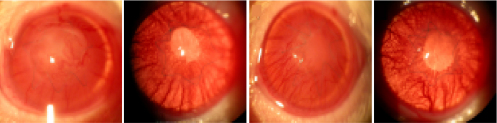
Demonstrative cases on slit-lamp examination 21 days after transplantation. Control corneas receiving either topical (**A**) or SC (**C**) denatured treatment were fully rejected with a maximum clinical score where clear grafts could be observed after topical (**B**) or SC (**D**) administration of active anti-VEGF therapy.

On Day 21 before the BN rats were sacrificed, all those in the control groups (G1 and G3) experienced corneal rejection, while three grafts (50%) and four grafts (66.67%) were rejected in G2 and G4, respectively. The treated groups experienced significantly less rejection than their respective control groups (G1 versus G2, p<0.005; G3 versus G4, p<0.05; Figure 1).

**Figure 2:**
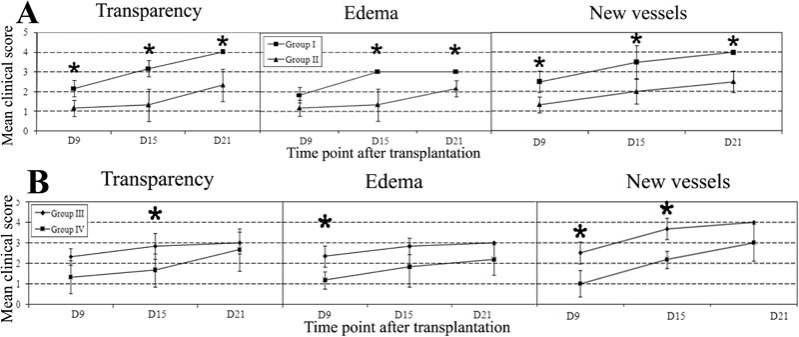
Mean clinical corneal scores observed after treatments versus matched controls. Mean clinical scores (±SEM) were higher in control animals treated with denatured SC injections (**A**, square line) compared to those treated with SC injections of active rat anti-VEGF (**A**, triangle line) for the three parameters assessed at every time point. With drop administration (**B**), the scores were also higher for controls (**B**, lozenge line), but not significantly after 21 days. The asterisk represents a p<0.05 statistical significance.

The MCSs for transparency, edema, and neovascularization are illustrated at Day 9, Day 15, and Day 21 after transplantation in Figure 2A,B. Even when there was no statistical significance; the MCSs were always lower for all assessed parameters in the treated groups compared to their related control groups. At each time point, the MCSs for neovascularization were significantly lower in the treated groups compared to the control groups, except at the end of the experiment for the topically treated group (G3 versus G4 at D21, p>0.05). Compared to the control group, edema in the SC treated group differed significantly only after Day 15. Conversely, whether topically treated buttons displayed significantly more edema during the acute phase of rejection at Day 9, no statistical difference was found at later time points. Transparency MCSs reached a statistical difference at every time point for SC injected eyes, while eyes treated with drops only differed at Day 15.

### Neovascularized area of corneal buttons

**Figure 3:**
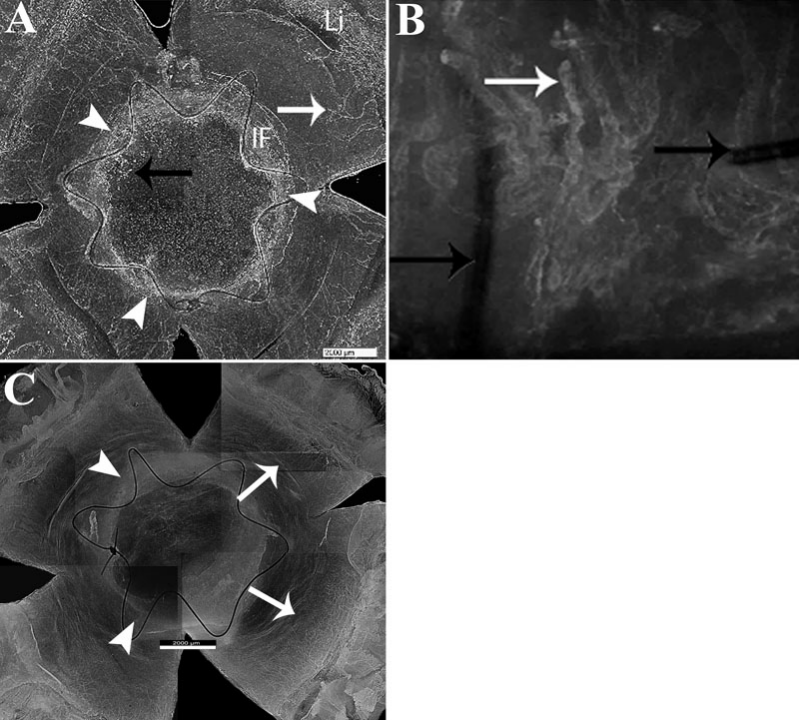
Flat mounted corneas stained for new vessels 21 days after allotransplantation. Allografted cornea treated with inactivated (**A**, **B**) or active (**C**) rat anti-VEGF antibody and flat mounted at Day 21; new vessels stained with lectin arise from the limbus toward the grafted area (**A**, **B**, **C**; white arrows**).** The progression of new vessels crossed the trephination line (**A**; arrow heads) across the stroma of the button (**A**, black arrow) between the strand of the running suture (**B**, black arrow), securing the corneal graft to the recipient bed. It was limited to the recipient area in treated animals (**C**; white arrows). Li, limbus; IF, interface between donor and recipient (**A**, **C**; arrowheads,). Scale Bar=2200 µm. Magnification, **A**, **C** 20×, **B** 100×.

Total areas and neovascularized areas of corneas are displayed in Table 1. The total mean areas of corneas (recipient cornea and button) did not significantly differ across groups (p>0.05).After SC injection with anti-VEGF (G1), the mean neovascularized area was 35.66% of the total mean cornea area compared to a mean neovascularized area of 57.82% after SC injection with denatured anti-VEGF (G2; p<0.005; Figure 3). After topical treatment (G3), the mean neovascularized area was 43.08% of the mean corneal area compared to 57.38% for the related controls (G4; p<0.005; Figure 3 and Table 1). Interestingly, the neovascularized area was significantly lower in the SC treated group than in the anti-VEGF drops group (p=0.01).

**Table 1:** Morphometric quantification of the neovascularized corneal area of flat mounted corneas from treated groups (G2=SC anti-VEGF and G4=topical anti-VEGF) and their relative controls (G1=SC inactivated anti-VEGF and G3=topical inactivated anti-VEGF).

**Groups of animals (G)**	**Total corneal (area mm^2^±SEM)**	**p value**	**Neovascularized corneal (area mm^2^±SEM)**	**Ratio (%)**	**p value**	**p value**
G1	113.05±3	>0.05	65.37±3.15	57.82	0.0039	0.01
G2	116.5±1.72	>0.05	41.56±3.62	35.67	0.0039	0.01
G3	114.23±1.89	>0.05	65.55±3.53	57.38	0.0039	0.01
G4	113.82±2.91	>0.05	49.03±3.94	43.07	0.0039	0.01

A p value of <0.05 represents a significant difference.

## Discussion

Pre-existing or secondary neovascularization predisposes to graft rejection after corneal allogeneic transplantation [27,28]. Neovessels arise from the limbus toward alloantigeneic tissue, i.e., the button, under the influence of pro-inflammatory cytokines produced by sensitized immune active cells [15,29]. Vascular endothelial growth factors and their receptors have been demonstrated to play a critical role in corneal neovascularization in both humans and in animal models [29-34]. Conversely, anti-VEGF therapy effectively inhibits corneal neovascularization in several rabbit models [12] as well as in patients [35].

In our study, we have chosen to administer a rat anti-VEGF specific antibody to optimally match with the animal model. Bock et al. demonstrated that humanized anti-VEGF antibodies (i.e., bevacizumab) bind rat VEGF-A with less specificity than humanized VEGF-A, and dissociate more rapidly because of a lack of specificity [20]. It was therefore possible to administer lower doses and concentrations of anti-VEGF antibody (0.6 µg/kg and 10 µg/ml) than in other studies (usually 5 mg/kg and 4 mg/ml) [20,36]. Increasing species specificity in experiments might prevent dose-dependent side effects or unspecific events while preserving the same efficacy. It is also likely that humanized anti-VEGF (Fab), while being less specific, is capable of interacting with cytokines other than VEGF itself. If so, they would potentially act through non-VEGF-mediated pathways. It is therefore more accurate to conclude that anti-VEGF treatment is effective through the VEGF-mediated pathway when the considered antibodies match animal species. Vascular endothelial growth factor levels in the time course of corneal graft rejection are of interest, as are other cytokine kinetics, while neutralizing VEGF. However, this paper was designed to compare two different routes of administering a VEGF neutralizing antibody specific to the rat VEGF in a rat model of corneal graft rejection in hopes of helping clinicians.

For proliferative diabetic retinopathy, VEGF levels were measured in the range of 500 pg/ml to 1 ng/ml and the minimal levels of neutralizing ranibizumab were evaluated at 10 µg/ml (the levels found at four weeks after injection), not necessarily correlating well with VEGF levels [37-39]. Hence, the observed clinical effect of anti-VEGF antibodies, although already semi-quantified by immunofluorescence in other studies [40,41], does not correlate well with VEGF levels in the ocular media or tissues. This is probably because the balance between VEGF and soluble VEGF receptors or other cytokines would influence the clinical results. Therefore, we think the clinical effect of two routes of administration is of interest for clinicians who use bevacizumab in cases of graft rejection without the benefit of reliable preclinical studies to guide their practice [19,42-45].

Both drop instillations and SC injections are relevant to target neovessels growing from limbal vessels [33,46]. While ocular instillation is less invasive and systemic rates are lower than those required for SC injections, drops also have lower bioavailability [47,48]. The mechanism that might explain how 150 kDa antibodies can effectively treat corneal neovascularization remains unclear. Antibody ocular penetration may be partially explained by the loss of corneal barrier integrity resulting from corneal surgery. The fact that topical instillation is less efficient in preventing neovessel growth after PK favors this hypothesis. Another explanation might be the limbal penetration of antibodies, as occurs with other peptides or F(abs) [49]. Direct transepithelial penetration of a 150 kDa protein through an intact cornea is unlikely to occur.

In our study, SC injections and drops had the same effect on the final rejection rate; however, SC injections decreased edema and the progression of new vessels more efficiently at later time points than at earlier ones, as confirmed by both MCS quantification and neovascular surface morphometry. Several conditions are necessary for an instilled drug to cross the epithelial barrier, such as polarity, hydrophilia or lipohilia, pH, osmolarity, and molecular weight [50]. The molecular weight of bevacizumab is 149 kDa, which is almost identical to the rat anti-VEGF neutralizing agent used in the present study. Even though the molecules are large, they are able to cross the healthy corneal epithelial barrier [51]. On the other hand, SC injections generate higher intraocular concentrations compared to systemic or topical administration [51] and contribute to the slower release of pharmacologic agents. After SC injection, anti-VEGF agents have proven to spread into the corneal stroma and to remain there for several days [12]. This may explain why neovascularization was more efficiently slowed down after SC injections rather than after drop instillations in our experiments.

Doctor et al. [35] recently observed that the previously neovascularized corneal area of eight eyes from seven patients treated with SC injections of bevacizumab was reduced due to ocular surface inflammatory diseases.

We tested anti-VEGF therapy on an animal model that mimicked corneal transplantations in humans without specific risk of rejection, such as preexisting neovascularization, to compare the two main local routes of administration commonly used to treat corneal diseases. To the best of our knowledge, a comparison of the efficacy between topical and SC administration has not been previously reported. Bachmann et al. used a high-risk graft rejection model to efficiently inhibit postoperative hemangiogenesis, lymphangiogenesis, the recruitment of antigen-presenting cells, and to improve corneal graft survival [16,52].

Animal models of corneal transplantation frequently use incorrect pathways to eventually mimic rejection. For example, models using separate sutures [53,54] might induce interfering inflammatory reactions [16] that are difficult to distinguish from true immune reactions. In the present model, we assumed that a continuous suture would limit irrelevant stimulations and minimize the unavoidable neovascularization that would occur when any persistent corneal foreign body, such as sutures, is present. Cursiefen et al. [53] observed that a total inhibition of corneal sutures induced neovascularization with a VEGF-A trap targeting VEGF and placental growth factor (PlGF). Using the same pathway, we only observed partial inhibition. This could argue against a similar effect of VEGF-A on neovessel induction during the inflammatory (foreign body) or alloimmune process.

Our results show that SC anti-VEGF injections decreased corneal neovascularization and increased the graft survival rate, reducing the neovascularized area by more than 20% (p<0.005). Topical treatment reduced it by approximately 15% compared to the control group (p<0.005) from Day 9 after the graft surgery until sacrifice. Thus, both routes seem to reduce the progression of corneal vascularization. However, combining the slow release effect and targeting anti-VEGF therapy to the precise location of early neovascularization during the rejection process (the limbus), SC administration seems more efficient than simple drops. The slight difference observed between the SC injection and topically treated groups in reducing neovascularization might also reflect higher volumes of anti-VEGF administered under the conjunctiva, which could act as a reservoir, gradually releasing drugs and permitting higher absorption by tissues. We also assume that immediate post-operative SC injection also limited the high amount of proinflammatory cytokines (TNF- α, IL-1) and growth factors (TGF-α, TGF-β, b-FGF, PDGF, and VEGF) produced by corneal fibroblasts migrating toward the wound at the early stage of wound healing (about 4 days in rats), thus possibly contributing to a non-immune proangiogenic environment.

Although significant, we only observed a slowdown in neovascular progression rather than a complete inhibition for the treated animals. This partial effect might be due to our treatment, which mainly targeted VEGF A, with less than 2% inhibiting cross-reactions with VEGF-B, C, and D. No antibody is currently available for rats to inhibit the VEGFR-3 lymphangiogenesis pathway. In a mouse model, Lyve-1 antibodies against VEGFR-3 inhibited new lymphatic vessels and dramatically limited graft rejection [17]. Furthermore, many other growth factors contribute to the graft rejection process, such as PlGF [55], TGF-α, TGF-β, bFGF, PDGF, or other VEGF independent factors such as IL-1β, IL- 6, or TNF [56]. To totally inhibit new vessels by acting on a single pathway therefore seems difficult. It is likely, however, that the global neovascularization process would not be affected only by VEGF inhibition. Hopefully, a therapeutic combination targeting the relevant cytokine group, possibly associated with additional methods such as gene therapy, would be more efficient.

The MCSs did not differ between treated and control animals, although they were slightly but constantly lower in the treated groups. This result suggests a possible inhibition that would not have been noticeable due to a lack of statistical power. A 90% statistical power combined with a 25% initial maximum risk (risk to obtain no spontaneous rejection in our model) would reveal a 135% relative risk reduction for rejection. In other words, the treatment should have been extremely efficient and the results should have been particularly clear cut, reaching statistical significance among groups of six animals. On the other hand, treated animals should be operated on the same day as their relative controls to prevent any procedural bias from interfering with clinical observations and outcomes. However, performing surgery on more than 12 usable animals in a single day would have rendered the experiment design unrealistic.

We repeated SC injections every 3 days, with low anti-VEGF concentrations (10 µg/ml compared to a range from 4 to 25 mg/ml for the usual concentration). We did not administer injections on a daily basis to limit local trauma and prevent local inflammation from interfering with the natural rejection process. Further work should certainly assess the optimal frequency for injections to obtain clinical efficiency. The optimal frequency for administering drops should also be explored as it is possible that more instillations would reduce the rejection rate or reduce the neovascularized area.

We attribute the relative graft survival observed after VEGF inhibition to a combination of mechanisms. The alloimune response occurring in corneal transplantation mainly involves T lymphocytes and antigen-presenting cells. The latter is thought to be driven to allogeneic corneal tissue through hematic and lymphatic vessels, growing from the host limbus under VEGF cytokine stimulation. By blocking VEGFR 2 and 3 pathways, anti-VEGF drugs inhibit the afferent route of immune response, which is critical for the sensitization of antigen-presenting cells. Additionally, VEGF inhibition directly affects the recruitment of inflammatory cells in the cornea [17], where neutrophilic polynuclear and macrophages are retained [57]. However, VEGF is not entirely inhibited because immunocompetent cells also produce hemangiogenic and lymphangiogenic growth factors [16,53].

Our observations are limited to rats. Species differences must be taken in account to apply the present results to human corneal transplantation. In a murine model, graft rejection is faster (delays of 4–10 days have been noted in previously immunized people), and both wound healing and immune reactions are exacerbated. Endothelial division is possible and the distance between graft and limbus differs. However, we can suggest some conclusions. From a histological and embryological point of view, tissues and observations are rather similar for both human and rat cornea organoculture [58,59].

According to our results, a specific to species anti-VEGF antibody is efficient for partial inhibition of corneal neovascularization. It increases the corneal graft survival rate in a rat model, but statistically significant results were only reached for the SC route of administration. Our experimental observations corroborate previous observations concerning the control of corneal neovascularization in humans. More experiments are needed to answer the key questions, which are how much VEGF is expressed in the cornea during the rejection time course and how much should be neutralized. How much the anti-VEGF antibodies administered by the two methods of administration are eventually existing in the target tissues is another interesting point. In the treatment of exsudative AMD and proliferative diabetic retinopathy, bevacizumab (Avastin®) is successfully used intra-oculary to control retinal and choroidal neovascularization; it has a low rate of side effects. If our results are confirmed in humans, the use of this simple cost-effective treatment could be of major importance for patients with a high risk of graft rejection. In conclusion, the present results are encouraging and support the further use of anti-VEGF in human clinical practice to treat corneal graft rejection using the SC route of administration.
